# Smooth Extubation Techniques in Pediatric Patients After LeFort I Osteotomy

**DOI:** 10.7759/cureus.14659

**Published:** 2021-04-24

**Authors:** Teresa Anabel Lucín Yagual, Sócrates Marcelo Vivanco Murillo, Nataly Vanessa Espinoza Daquilema, Raisa Stefanía Mariscal García, Daniel Fernando Dick Paredes

**Affiliations:** 1 Anesthesiology and Reanimation, Hospital General Guasmo Sur, Guayaquil, ECU; 2 Oral and Maxillofacial Surgery, Hospital General Guasmo Sur, Guayaquil, ECU; 3 Pediatric Medicine, Hospital General Guasmo Sur, Guayaquil, ECU; 4 Emergency, Hospital Matilde Hidalgo de Procel, Guayaquil, ECU

**Keywords:** smooth extubation, mid-facial hipoplasia, difficult airway, difficult extubation

## Abstract

The anesthetic approach to patients with facial deformities, such as midface hypoplasia (MFH), is complex and requires coordinated work with the surgical team. These patients may have a difficult airway (DA), and hence special considerations must be taken from the anesthetic point of view, and several options have been described by the American Society of Anesthesiology (ASA) related to this. Multiple methods have been described for intubation and maintenance; for extubation in pediatric patients, there are no defined guidelines. Extubation can be performed under deep anesthesia or with the patient awake, taking special considerations by keeping their condition in mind; these approaches have shown varying results. Favorable outcomes have been observed in the literature and personal experiences with smooth extubation techniques in patients at a high risk of reintubation, such as those with dentofacial deformities and the pediatric population.

A 15-year-old girl with a diagnosis of severe malar hypoplasia associated with a cleft lip (CL) was admitted to our hospital. She had a history of previous surgeries and had persistent functional disorders, for which surgical placement of facial distractors was scheduled. For the anesthetic approach, a balanced general anesthesia option was chosen. The use of a video laryngoscope was determined to be the proper choice for DA, with the fixation of the oral endotracheal tube (OETT) in a caudal direction, and with mechanical-ventilator settings appropriate for the patient's age. Deep extubation with smooth extubation techniques was performed successfully. No anesthetic complications were observed in this case.

## Introduction

Midface hypoplasia (MFH) is a dentofacial malformation (DFM) associated with several congenital malformations including cleft lip (CL) and cleft palate (CP). CL is the most common congenital malformation among craniofacial anomalies. The incidence rate of CL in pediatric patients is 3.5 million worldwide. It is associated with various degrees of skeletal malocclusions, mostly class III malocclusion (MO CIII), severe underbite, unilateral or bilateral crossbite, alterations of the maxillary dental arch in a sagittal, vertical, and transverse direction, increased Spee curve, multiple dental agenesis, oronasal communication, and residual fissure. Furthermore, the mandible tends to rotate upward and forward with the subsequent decrease in the vertical dimension, loss of anterior facial height, pseudoprognathism, and alterations in phonation [1,2].

DFMs can be corrected with orthognathic surgical procedures, such as the LeFort I osteotomy, mandibular ramus sagittal osteotomy, and combined surgery [1,3]. The LeFort I osteotomy is the most commonly used orthognathic surgical method and consists of an intermaxillary ligation, with an external arch that is fixed to the skull with multiple screws, an intraoral splint whose fixation to the external traction hooks is intranasal and with surgical wires [4]. Following a LeFort I osteotomy, airway alterations occur in patients, which are caused by the devices placed in the maxilla and nostrils, in addition to bleeding from the manipulated bone segments [3,4].

The pediatric age group is associated with congenital and acquired diseases, with repercussions in the airway and the intubation or ventilation process. A difficult airway (DA) can be expected based on the characteristics of the patient or not expected when it is difficult to recognize, being found when ventilating or intubating a patient [5,6]. The predictors of DA in pediatric patients are not specific, because they were not created for this age group [7]. Another important factor to consider is that the medical literature focuses mainly on the intubation process and there is little information on extubation, despite the fact that it is a highly important procedure as it could avoid unnecessary risks, complications, and poor outcomes [3,8].

Airway edema and laryngospasm account for 5% of the causes of death since they are the main complications reported in this type of surgery [3,8]. DA presents multiple challenges at the time of extubation; altered ventilation and previous endotracheal intubation are potential indicators of reintubation [9].

## Case presentation

**Figure 1:**
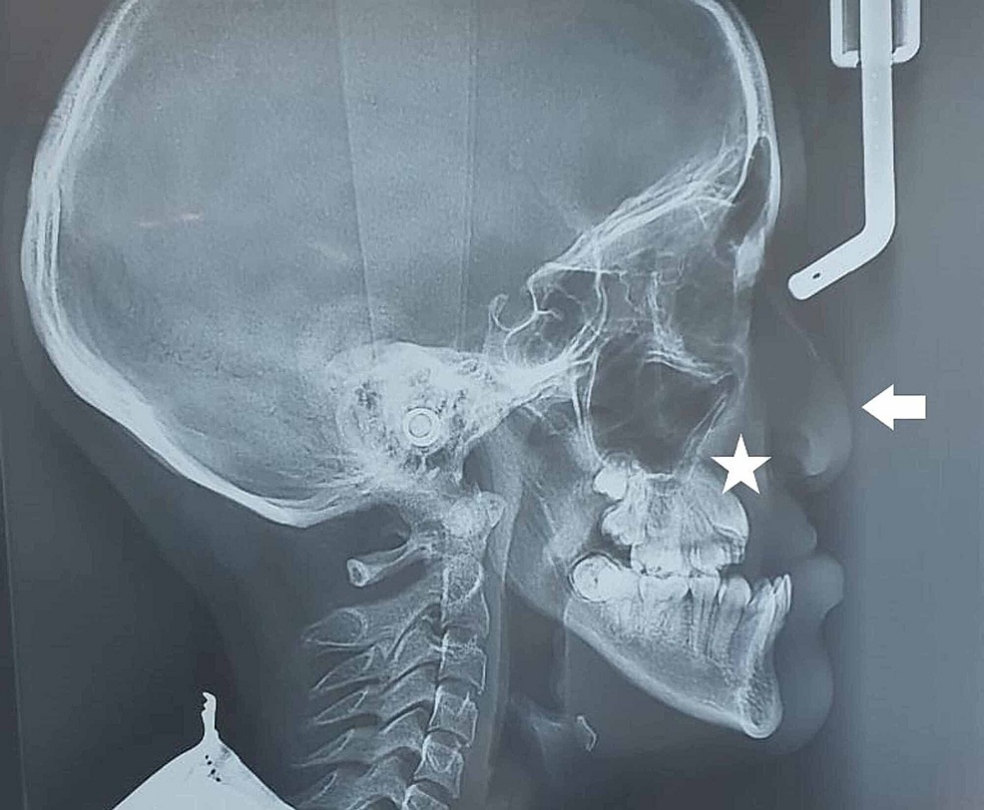
Lateral cephalic X-ray Mandibular prognathism was observed at the expense of maxillary hypoplasia (star). In soft tissues, there was drooping nasal tip (arrow); however, an unobstructed airway and preserved diameters were observed

A 15-year-old girl presented to the hospital with a diagnosis of MFH associated with CL and bilateral CP. She had a surgical history of cheiloplasty and palatoplasty when she was three months of age; both operations had been revised on multiple occasions. At the time of the surgery, she had presented transverse collapse of the maxilla, anterior crossbite, persistence of a bilateral nasoalveolar fistula, and mandibular prognathism. The patient had no chronic degenerative diseases or significant comorbidities. During the anesthetic evaluation of the airway, a hard palate cleft was found. Other parameters were found to be adequate, and her laboratory tests were normal. The X-ray showed the sequelae of CL and CP (Figure 1).

Anesthetic process

**Figure 2:**
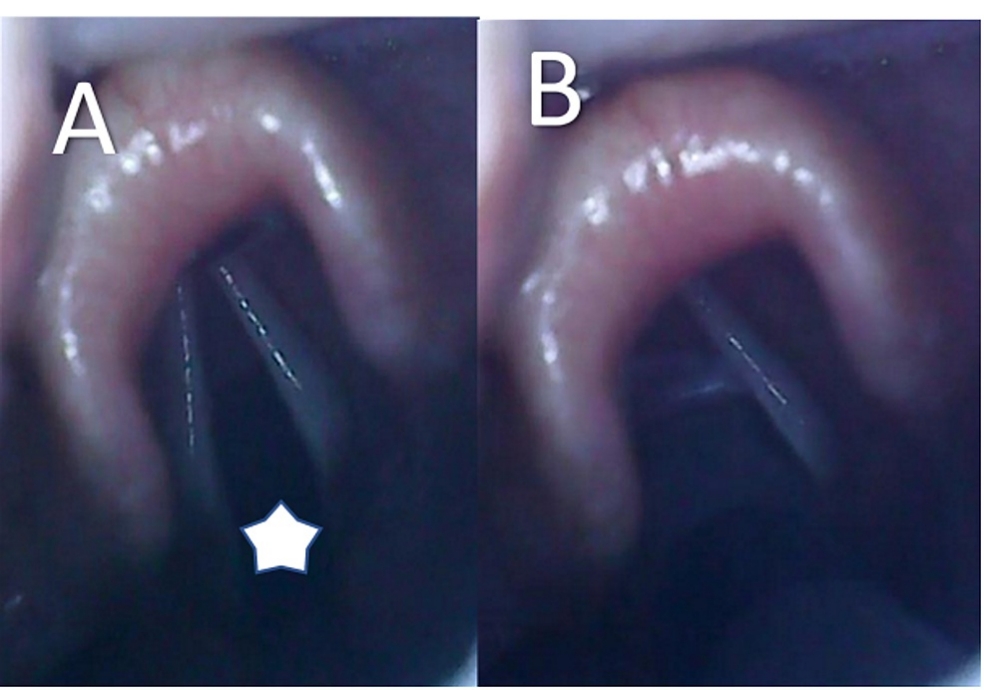
Videolaryngoscopy and intubation A: open glottis (star). B: video-assisted endotracheal tube introduction

A balanced general anesthesia approach was planned. Induction was performed with midazolam 3 mg, fentanyl 100 mcg, propofol 80 mg, and rocuronium 30 mg. The video-assisted laryngoscopy was performed, in which Cormack I was identified, and the procedure was continued with the introduction of an endotracheal tube (ETT) # 6, lubricated with 2% lidocaine gel and set at 18 cm and with the balloon insufflated with 5 cc of air (Figure 2). Caudal fixation of the tube was performed and eye protection with total occlusion was set. Invasive mechanical ventilation was pressure-assisted. For maintenance, sevoflurane 2% [minimum alveolar concentration (MAC): 1] and fentanyl 2 mcg/Kg/minute were administered.

Intraoperative care consisted of gastric protector, prophylaxis for nausea and vomiting, analgesia, and fibrinolytics. During the surgery, the vital signs were stable, maintaining permissive hypotension. Parenteral fluids were managed according to the hydration formula of Holliday and Segar (4-2-1 rule), with a total of 1,424 ml, out of which approximately 1,400 ml of sodium chloride 0.9% were administered during the four hours of surgery. Hydrocortisone 100 mg was used as an airway spasm prophylactic. There was scant bleeding during the procedure.

Emergence

After the mandibular traction dispositive was set, extubation maneuvers were started. No reversal of neuromuscular blockade was performed, based on drug half-life (surgical time was four hours) and the fact that no rescue dose of anesthetic drugs was used during the intraoperative time.

Deep extubation was planned due to MAC AWAKE (alveolar concentration on awakening) of 0.3 and since the fentanyl infusion had stopped 45 minutes before extubation; smooth extubation techniques were used. We made sure that it complied with the criteria of conventional extubation and with stable hemodynamic parameters during and after extubation. In case of complications or the need for reintubation, the emergency intubation equipment was available; in case the airway access was not fast enough, a surgeon was present so that the equipment could be removed, which was not necessary.

The patient regained consciousness after one minute; she woke up, obeyed orders, opened her mouth, presented a sustained head-lift, and denied pain, nausea, or vomiting. She was then transferred to the post-anesthetic recovery area where she was monitored for two hours, with no hemodynamic alterations. Later, she was shifted to the hospitalization area, where she remained for 72 hours, accompanied by her mother.

## Discussion

**Figure 3:**
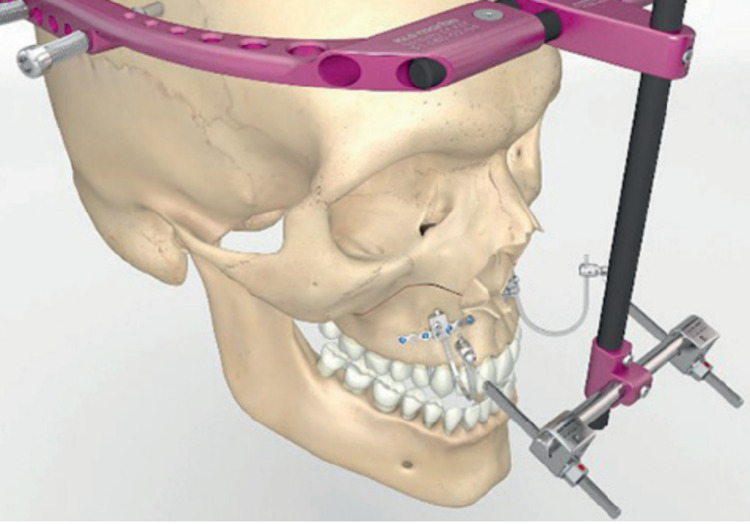
Rigid external distraction with intranasal bone-borne traction Hooks for midfacial hypoplasia Source: KLS Martin Group

Our patient presented postoperative sequelae due to correction of CL, characterized by MFH. Her face, as seen in the profile, was concave - flattened with a wide nasal base, which preceded malocclusion and was classified as class III, with an anterior crossbite. The literature indicates that the best surgical option for these types of cases is the LeFort I osteotomy (Figure 3) [1].

Studies that deal with the anesthetic considerations of this procedure have highlighted the importance of establishing strategies to prevent complications of airway management, which would help lead to successful extubation. In pediatric patients, these factors acquire greater significance due to anatomical differences and different responses to stimuli that are characteristic of this population. These factors directly influence the appearance of post-surgical complications, which would necessitate reintubation in the worst-case scenario, in addition to having an impact on recovery, safety, comfort, and hospital stay [8,10,11].

The decision-making process and elaboration of the strategic plan for this type of surgery represent a high anesthetic and surgical risk, based mainly on the surgeon and anesthesiologist working in the airway. For this reason, it should ideally comprise multidisciplinary management at the different stages of anesthesia: pre-surgical, trans-surgical, and immediate postoperative [2,7].

Currently, there is scarce information in the literature on the extubation technique in the management of the airways. The Difficult Airway Society (DAS) developed a guide for the management of tracheal extubation in 2012, but it failed to establish a difference between successful extubation (patients who tolerate the removal of the ETT) and smooth extubation. In this guide, extubation management was proposed to be performed in stages: planning the extubation, preparing for extubation, performing extubation, and post-extubation care [12-14].

Taking this literature as a premise, along with the anatomical changes produced by the surgical procedure, we decided to use smooth extubation, which decreased the risk of hemodynamic complications, alterations or irritations of the airway, difficulty in ventilation of the patient, and the need for reintubation [9,15].

Smooth extubation is a term that has been used for a few years in the medical literature, and its purpose is to avoid any physiological response that may lead to adverse extubation results. It has been shown that smooth extubation prevents hemodynamic and respiratory responses such as hypertension, tachycardia, arrhythmias, cough, laryngospasm, myocardial ischemia, and increased intracranial and intraocular pressures [9]. One of the advantages of smooth extubation includes the use of pharmacological aid that includes drugs that decrease the incidence of odynophagia, cough, and laryngeal reflexes, as well as drugs that provide a degree of sedation, such as the ones used in pain management. In our patient, we employed a multimodal strategy, including intravenous opioids, local anesthetics, and corticosteroids. The opioid used in this procedure was fentanyl, due to the limitations experienced at the time, although the literature mentions greater benefits with the use of dexmedetomidine and IV remifentanil; in this case, one of the challenges was adapting to the drug used, considering its half-life and pharmacokinetics [9,15].

Smooth extubation is always performed by preserving the patient's airway, using pre-oxygenation maneuvers with 100% FiO_2_ and Guedel cannula, and medications among which ketamine, dexmedetomidine, remifentanil, fentanyl, IV and topical lidocaine, and corticosteroids stand out [9]. These techniques have been observed to be used in daily practice, but it depends on the preferences of the anesthesiologist or how familiar the anesthesiologist is with these maneuvers. The cough reflex, movements during extubation, and holding the breath or laryngospasm are considered indicators of failed extubation [9,12].

In our patient, the aforementioned maneuver enabled her to have successful extubation, and then, after extubation, no supplemental oxygen, rescue medication, or ventilatory assistance techniques were required in addition to adequate pain management and maintenance of stable vital parameters.

Failed extubation may not be apparent immediately upon the removal of the ETT; therefore, during the transfer of the patient, monitoring, and emergency reintubation if required, should be ensured; the semi-flower position and the nasal cannula have been suggested for the first hours with 100% O_2_ [10]. 

Based on the DAS guidelines, there had been other elaborate guidelines such as the ones from the All India Difficult Airway Association (2016) and the French Society of Anesthesia and Reanimation [Société Française d'Anesthésie et de Réanimation (SFAR)]. However, none of the algorithms found in these guidelines focus on the pediatric population [12,14].

## Conclusions

Smooth extubation techniques are a great method for the management of patients at higher risk of complications, such as pediatric patients with DAs, since they provide a good safety margin and reduce the risk of reintubation and further problems. However, more studies are required to establish proper protocols for its use and ensure better expectations/outcomes for the patients, especially pediatric patients who face their own unique challenges.
